# Deep Reinforcement Learning-Based Traffic Signal Control Using High-Resolution Event-Based Data

**DOI:** 10.3390/e21080744

**Published:** 2019-07-29

**Authors:** Song Wang, Xu Xie, Kedi Huang, Junjie Zeng, Zimin Cai

**Affiliations:** College of Systems Engineering, National University of Defense Technology, Changsha 410073, China

**Keywords:** traffic signal control, deep reinforcement learning, high-resolution data, event-based data, double dueling deep Q network

## Abstract

Reinforcement learning (RL)-based traffic signal control has been proven to have great potential in alleviating traffic congestion. The state definition, which is a key element in RL-based traffic signal control, plays a vital role. However, the data used for state definition in the literature are either coarse or difficult to measure directly using the prevailing detection systems for signal control. This paper proposes a deep reinforcement learning-based traffic signal control method which uses high-resolution event-based data, aiming to achieve cost-effective and efficient adaptive traffic signal control. High-resolution event-based data, which records the time when each vehicle-detector actuation/de-actuation event occurs, is informative and can be collected directly from vehicle-actuated detectors (e.g., inductive loops) with current technologies. Given the event-based data, deep learning techniques are employed to automatically extract useful features for traffic signal control. The proposed method is benchmarked with two commonly used traffic signal control strategies, i.e., the fixed-time control strategy and the actuated control strategy, and experimental results reveal that the proposed method significantly outperforms the commonly used control strategies.

## 1. Introduction

Traffic congestion, which causes extra travel delay, enormous economic waste, and excess vehicular emission [1], has become a problem in many cities all over the world. Effective traffic management and control are crucial for relieving the deteriorating traffic situation, especially in the context of large-scale construction of road infrastructures being restricted by limited space and funds. Traffic signal control, which is one of the key traffic control strategies, has significant potential to improve traffic performance by optimizing the use of intersections.

Conventional fixed-time and actuated signal control strategies are inefficient since they have no or limited ability to handle dynamic changes of traffic demands [2,3]. Consequently, adaptive traffic signal control, which adjusts the control parameters (e.g., the cycle length, phase splits, and the offset) based on real-time traffic conditions [4], are proposed with aims to perform optimized signal operations. In recent years, reinforcement learning (RL), a type of algorithm which can learn from experience [5], is increasingly applied in the design of adaptive signal controllers.

### 1.1. Reinforcement Learning-Based Traffic Signal Control

In a RL-based traffic signal control (RLTSC) system, the traffic signal controller can be modeled as an intelligent agent interacting with the traffic environment. Generally, the agent has no prior knowledge about its environment (intersection) and dynamically learns from interactions. Observing a traffic condition (state), the agent inspects available actions and chooses the action based on its experience (control policy). After performing the action, the traffic environment responds to the agent with a scalar reward signal, which reveals the quality of the selected action with respect to its objective. Using the received reward signal, the agent updates its experience (control policy) to optimize the accumulated rewards in the long run [6]. RLTSC is a novel approach to handle the stochastic traffic environment because of its autonomic and intelligent characteristics, including the ability of self-learning to continually refine its control policy without any prior knowledge, the adaptability to fluctuated traffic conditions, and the flexibility to customize its optimization objective [5,6].

Designing a RLTSC system mainly considers five elements: the state definition of the environment, action space definition, action selection method, reward function definition, and learning algorithm [7]. Among others the state definition determines how the traffic environment is represented to the agent, and the reward function specifies the objective of the agent [5,8]. The state and reward are all observations that the agent can receive from the traffic environment, based on which the agent learns and optimizes its control policy. Therefore, they play pivotal roles in the development of RLTSC systems. A variety of traffic data have been employed to represent the traffic state and reward in RLTSC research.

Abdulhai et al. [9] applied Q-learning to control the traffic signals at an isolated intersection. They selected the queue lengths on the approaching links and the elapsed phase time as the traffic state, and defined the reward with the total delay of vehicles incurred between consecutive decision points. Test results show that the Q-learning controller is superior to the pretimed signal timing under variable traffic conditions. From a practical point of view, it is of great importance that the observations can be readily measured using prevailing detection systems for signal control (e.g., loop detectors), since precise estimates are usually hard to obtain, and deploying a sophisticated detection system is considered to be expensive [10,11,12,13]. Richter et al. [10] represented the traffic state using the states of traffic signals (the cycle length, phase splits, and current phase and its duration) and the statuses of detectors near the stop lines, and calculated the reward signal with the number of vehicles which entered the intersection over the time step. They employed the natural actor-critic learning algorithm to optimize traffic signals. Prashanth and Bhatnagar [11] proposed a feature-based state representations in a Q-learning-based traffic signal control algorithm. In this algorithm, queue lengths and the elapsed time of each lane in the traffic network were not directly used in the state definition; instead, the congestion levels (low, medium, or high) and graded elapsed times (blow or above a threshold) of lanes were defined as the state. Similarly, Prabuchandran et al. [12] characterized queue size as low, medium, or high via two sensors deployed on each lane. They defined the segregated queue lengths along with the index of next green phase as the state of the environment, and used the segregated queue lengths of neighboring junctions to calculate the reward signal. Jin and Ma [13] employed SARSA(λ) learning algorithm to develop an adaptive group-based signal control system, in which each signal group is modeled as an agent. They used the information of the time gap and occupancy, both of which can be obtained from the loop detectors directly, along with the phase status and elapsed green time to represent the traffic state. However, these data, such as the status of detectors (occupied or idle), the lowest time gap, and the congestion level, are coarse and miss some critical features for signal control.

To capture dynamics of the traffic environment as fully as possible and achieve more efficient signal control policies, deep reinforcement learning, a technique which combines reinforcement learning and deep learning, has been increasingly employed in developing adaptive signal control system [14,15,16,17]. Li et al. [14] integrated Q-learning with the deep stacked auto-encoders (SAE) neural network for designing adaptive signal timing plans. The traffic state and reward were defined based on queue lengths of incoming lanes. Simulation results reveal that the deep reinforcement learning method notably outperforms traditional reinforcement learning-based approaches. Some researchers [15,16] argued that human-crafted features such as the queue length ignore some useful traffic information, and state data should contain as much traffic information as possible to derive the optimal control policy. For obtaining as much useful information as possible, Gao et al. [16] used deep convolutional neural network to extract machine-crafted features from raw traffic data (position and speed of vehicles, and traffic signal state). The change of vehicle staying time over the green light interval was considered to be the reward. Liang et al [17] divided the whole intersection into small square-shape grids, and used the position and speed information of vehicles to construct the traffic state. The increment in cumulative waiting time over the cycle was considered to be the reward. To handle the challenges from the huge state space, a double dueling deep Q network (3DQN) with prioritized experience replay was proposed to learn the control policy. It should be noted that although these detailed data, such as the position and speed of vehicle and vehicular waiting time, contain abundant information about the traffic condition, it is quite difficult to collect them in current traffic engineering practice [5,13].

### 1.2. Contribution and Organization of This Paper

In this paper, we propose a deep reinforcement learning-based traffic signal control method which uses high-resolution event-based data, aiming to achieve a cost-effective and efficient adaptive traffic signal control system. High-resolution event-based data (referred to as event data in this paper), which keeps track of vehicle passage and presence by recording vehicle-detector actuation/de-actuation events, contains much more useful information compared with traditional aggregated data [18], and more importantly, it can be easily collected from vehicle-actuated detectors (e.g., inductive loops) with current technologies [19,20]. In the proposed RLTSC system, all observations that are used to define the traffic state and reward signal can be directly measured using the prevailing detectors, which makes the system completely deployable. Giving the event-based data, an encoding method is put forward to define the traffic state and deep learning techniques are employed to automatically extract useful features for traffic signal control, which gives the controller outstanding performance. The proposed method is validated on a microscopic traffic simulator, and benchmarked with two commonly used traffic signal control strategies, i.e., the fixed-time control strategy and the actuated control strategy. The experimental results show that the proposed method outperforms the commonly used control strategies.

The rest of this paper is organized as follows. Section 2 depicts the problem of traffic signal control using the framework of Markov decision processes (MDPs). Section 3 defines key RL elements for traffic signal control based on event data. Section 4 introduces the deep reinforcement learning approach for traffic signal control. Simulation experiments for training and evaluating the proposed method are presented in Section 5. Finally, the paper is concluded in Section 6.

## 2. Traffic Signal Control as a Markov Decision Process

In a signalized intersection, vehicle streams are governed by traffic signals (using green, yellow and red indications) to avoid movement conflicts. A phase refers to a state of the signals during which a particular set of non-conflicting traffic streams have right of way [21,22]. The objective of signal timings is to move vehicles through an intersection safely and efficiently by allocating right of way to the various streams, and there are many signal timing parameters (e.g., the phase sequence and phase durations) that affect traffic efficiency [23]. Adaptive traffic signal controllers attempt to adjust signal timing settings online in response to current traffic conditions, thus improve the traffic performance at intersections. Traffic signal control problem can be formulated by MDPs, which is an essential element underlying reinforcement learning [24].

**Figure 1:**
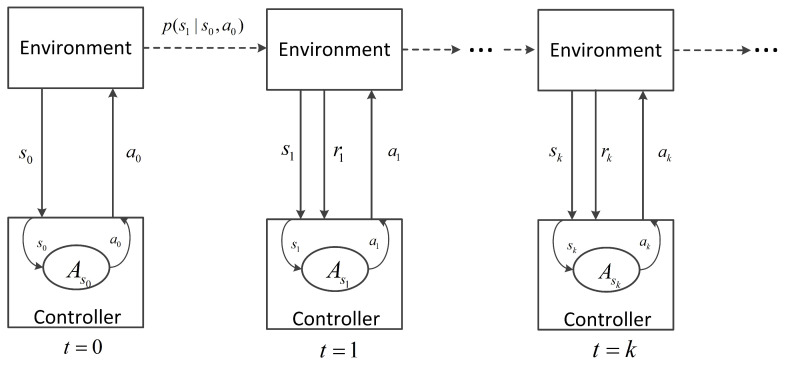
The interaction between the signal controller and the traffic environment.

In the framework of MDPs, the signal controller interacts with the traffic environment as follows: at the decision step *k*, the controller first senses the environment and obtains the state $s_{k}$, based on which the controller selects an action $a_{k}$ from the allowable action set $A_{s_{k}}$. Then, the controller executes $a_{k}$. As a result, of the action execution, the traffic environment evolves to new state $s_{k+1}$ with a probability of $p(s_{k+1}|s_{k},a_{k})$ and feeds back a scalar reward $r_{k+1}$ to the controller at the next decision step k+1. This process is iterated as illustrated in Figure 1.

Mathematically, the traffic signal control problem can be modeled by the following ingredients [25]:

- *S*: the state space, which consists of all possible traffic states, $s_{k}\in S,k=0,1,\ldots$;
- *A*: the action space, $a_{k}\in A_{s_{k}},A=\cup_{s_{k}\in S}A_{s_{k}},k=0,1,\ldots$;
- $r(s_{k},a_{k})$: S×A→R, the reward function;
- $p(s_{k+1}|s_{k},a_{k})$: S×A×S→R, the transition probability function.

Given a traffic state *s*, the controller selects an action *a* following a control policy π:S→A, which maps states to actions. With respect to a policy, state-value and action-value functions are defined. The state value of state *s* following policy π, donated as $v_{\pi }\left(s\right)$, is defined as:

(1) $$v_{\pi }\left(s\right)=E\left[\sum_{n=0}^{\infty }\gamma^{n}r\left(s_{k+n},\pi \left(s_{k+n}\right)\right)|s_{k}=s\right]$$

where E[·] represents the expected value of a random variable; γ∈[0,1] is the discount factor, which determines the importance of future rewards. Thus, $v_{\pi }\left(s\right)$ is the expected discounted future rewards when the environment starts from state *s* and the controller selects the action based on policy π. Based on the definition of $v_{\pi }\left(s\right)$, the action-value of taking action *a* in state *s* and following policy π thereafter, donated as $q_{\pi }\left(s,a\right)$, is defined by

(2) $$q_{\pi }\left(s,a\right)=E\left[r\left(s,a\right)+\gamma v_{\pi }\left(s_{k+1}\right)|s_{k}=s,a_{k}=a\right]$$

The goal of the controller is to find an optimal control policy $\pi^{*}$ to obtain the maximized state-value function $v^{*}$, i.e.,

(3) $$\pi^{*}\left(s\right)=\underset{\pi }{\mathrm{argmax}}v_{\pi }\left(s\right),\forall s\in S$$

The optimal action-value function $q^{*}$ is the action-values under policy $\pi^{*}$ (i.e., $q^{*}\left(s,a\right)=q_{\pi^{*}}\left(s,a\right),\forall s\in S,a\in A$). Since the optimal policy always chooses the action which maximizes the action-value, the Bellman optimality equation for the optimal action-value function $q^{*}$ holds:

(4) $$q^{*}\left(s,a\right)=E\left[r\left(s,a\right)+\gamma \max_{a_{k+1}\in A_{s_{k+1}}}q^{*}\left(s_{k+1},a_{k+1}\right)|s_{k}=s,a_{k}=a\right],\forall s\in S,a\in A$$

## 3. Definitions of Key RL Elements for Traffic Signal Control

In this paper, a model-free reinforcement learning algorithm, which does not need any prior knowledge about dynamics of the traffic system (e.g., the state transition probabilities), is employed for traffic signal control. It adjusts the signal phase based on the real-time traffic data collected from the intersection. In this section, we first introduce the configuration of detectors which are used to collect event data. Then, we define the intersection state based on the event data, and depict the definitions of the action and reward.

### 3.1. Configuration of Detection System

**Figure 2:**
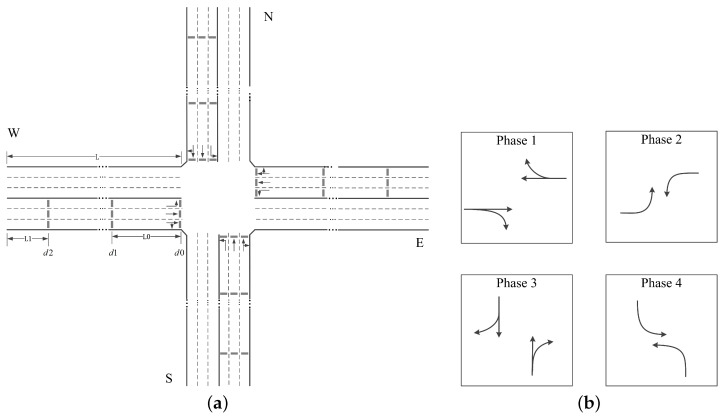
A typical 4-arms intersection. (**a**) The geometry and detection system at the intersection; (**b**) The signal phases at the intersection.

To obtain the traffic information required by the signal controller, three vehicle-actuated detectors (e.g., inductive loops) are configured for each lane approaching the intersection. Starting at the stop line, the first detector, donated as d0, is installed at the stop line, which is used to record the vehicle throughput. The next detector, donated as d1, is setback a distance of L0 from the stop line. It can reflect the traffic condition when no long queue is formed. The last detector, donated as d2, is placed near the entrance of the lane with a distance of L1. It is used to provide extra information especially when long queues occur. Figure 2a shows the detectors at a typical 4-arms intersection, where the length of each approaching road is *L* and all approaching roads have the same detection configuration. This detection system provides the event data for defining the intersection state and reward, which will be explained in following subsections.

### 3.2. State Definition

Traditional aggregated traffic data, such as the average occupancy and speed, loses much useful information for signal control. Therefore, we employ the informative event data to define the intersection state aiming at making full use of the available traffic information and achieving a better optimized traffic signal controller.

Specifically, we use the event data collected from the previous ΔT time interval to reflect the current state of the traffic at the intersection. Inspired by the definition of discrete traffic state encoding (DTSE) [15], a discrete time traffic state encoding (DTTSE) method is proposed to define the state using event data. In this method, ΔT is discretized into time steps of length dt. The dt should not be greater than the minimum time headway so that at most one vehicle-detector actuation event occurs at a detector during this dt interval. However, if dt is much smaller than the minimum time headway, it might lead to unnecessary computational cost.

**Figure 3:**
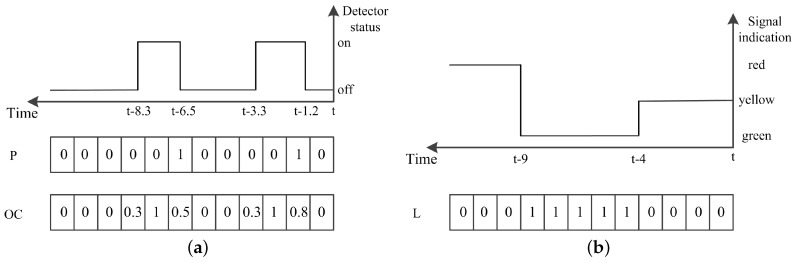
The illustration of discrete time traffic state encoding. (**a**) encoding the event data collected by detectors; (**b**) encoding the signal indication for a lane.

For each detector, two vectors are defined to record the vehicle-detector actuation events occurring on it in the previous ΔT interval. The first one *P* is a binary-valued vector, $P\in B^{\frac{\Delta T}{dt}}$, which represents the presence of the vehicle-detector actuation events or not in each discretized step, while the other vector OC, $OC\in R^{\frac{\Delta T}{dt}}$, records the occupancy in each step. Figure 3a illustrates this encoding via an example.

To retrieve the traffic state by using the detected data in a time period, the state of traffic signals during the period is an important factor to be considered. We record signals by storing the green indication for each lane. Specifically, for each discretized step, the ratio of the duration, when the signal is green, is stored. The signal state is donated by $L\in R^{\frac{\Delta T}{dt}}$ as is illustrated in Figure 3b.

We use the event data from detectors d1, d2 along with the signals states to define the intersection state. Assuming an intersection with *n* approaching lanes numbered from 1 to *n*, there are 2n encoded vectors for the event data from detectors d1, donated as $P1_{1}$,$OC1_{1}$ ..., $P1_{n}$, $OC1_{n}$, 2n encoded vectors for the event data from d2, donated as $P2_{1}$,$OC2_{1}$ ..., $P2_{n}$, $OC2_{n}$, and *n* encoded vectors for the signal state data, donated as $L_{1}$, ..., $L_{n}$. Since the state data is inputted to a convolutional neural network (CNN) (it is depicted in Section 4), we construct the intersection state by organizing these encoding vectors as a set of fixed-size matrices, donated as Mats, as follows.

$$Mats\left(2i\right)=\left[\begin{array}{c} OC1_{1}\left[\left(i-1\right)\frac{\delta t}{dt}:i\frac{\delta t}{dt}\right]\\ P1_{1}\left[\left(i-1\right)\frac{\delta t}{dt}:i\frac{\delta t}{dt}\right]\\ L_{1}\left[\left(i-1\right)\frac{\delta t}{dt}:i\frac{\delta t}{dt}\right]\\ ..\\ OC1_{n}\left[\left(i-1\right)\frac{\delta t}{dt}:i\frac{\delta t}{dt}\right]\\ P1_{n}\left[\left(i-1\right)\frac{\delta t}{dt}:i\frac{\delta t}{dt}\right]\\ L_{n}\left[\left(i-1\right)\frac{\delta t}{dt}:i\frac{\delta t}{dt}\right] \end{array}\right];Mats\left(2i+1\right)=\left[\begin{array}{c} OC2_{1}\left[\left(i-1\right)\frac{\delta t}{dt}:i\frac{\delta t}{dt}\right]\\ P2_{1}\left[\left(i-1\right)\frac{\delta t}{dt}:i\frac{\delta t}{dt}\right]\\ P1_{1}\left[\left(i-1\right)\frac{\delta t}{dt}:i\frac{\delta t}{dt}\right]\\ ..\\ OC2_{n}\left[\left(i-1\right)\frac{\delta t}{dt}:i\frac{\delta t}{dt}\right]\\ P2_{n}\left[\left(i-1\right)\frac{\delta t}{dt}:i\frac{\delta t}{dt}\right]\\ P1_{n}\left[\left(i-1\right)\frac{\delta t}{dt}:i\frac{\delta t}{dt}\right] \end{array}\right];\forall i=1,\ldots ,\frac{\Delta T}{\delta t}$$

where Mats(i) represents the *i*-th matrix; ΔT is divided into several periods with fixed-length δt, as the data in each period has respective features and contributions reflecting current traffic state. The encoded event data from d1 as well as the encoded signal states in each period are placed in a matrix, while the encoded event data from d2 along with presence vectors from d1 are organized into a matrix. The main motivation behind this organization way lays that filters are used to convolve with the respective input image (matrix) in CNN, leading to respectively extract the features of the event data from d1, d2 in each period.

In this paper, we set ΔT=60 seconds, dt=1 second and δt=20 seconds. For a typical intersection with 4 ways of 3 lanes such as the one in Figure 2a, the traffic state consists of 6 matrices with size 36×20.

### 3.3. Action Definition

In this research, we define the set of all feasible signal phases at the intersection as the action space *A*, and $A_{s}=A,\forall s\in S$. At each decision step, the controller selects a phase from *A*, then, actuates the phase and lasts for a time duration of $\tau_{g}$. This acyclic phase scheme makes the signal control highly flexible. In addition, considering the traffic safety at intersections, a yellow time of length $\tau_{y}$ is enforced, during which the running vehicles are cautioned to prepare to stop, before the traffic signals switch to another phase.

### 3.4. Reward Definition

Defining a proper reward function is very important for the RLTSC, since it evaluates the chosen actions and guides the optimizing direction. In this paper, we rely our reward on the number of vehicles entered the intersection and the waiting time of vehicles staying on detectors, both of which can be collected using the proposed detection system.

Specifically, we define the reward as

(5) $$r\left(s,a\right)=\frac{vn(s,a)}{sf_{t}\left(a\right)}-\alpha_{0}\sum_{i\in A}\frac{wait_{i,0}\left(s,a\right)}{sf_{w}\left(i\right)}-\alpha_{1}\sum_{i\in A}\frac{wait_{i,1}\left(s,a\right)}{sf_{w}\left(i\right)}$$

where vn(s,a) represents the number of vehicles entered the intersection during the time step, $wait_{i,0}\left(s,a\right)$ and $wait_{i,1}\left(s,a\right)$ represent the waiting time of vehicles collected by d0 and d1 on the lanes of phase *i* during the time step, respectively. Since the number of lanes and allowed turning streams in signal phases might be different, we introduce factors $sf_{t}\left(a\right)$ and $sf_{w}\left(a\right)$ for each action (i.e., signal phase) *a* in order to achieve more fair reward and better performance, and $\alpha_{0}$, $\alpha_{1}$ are trade-off coefficients. With this multi-objective reward signal, the proposed controller intends to maximize vehicle throughput and minimize the trip delay through learning.

## 4. Traffic Signal Control through Double Dueling Deep Q Network

Confronted with the raw traffic data and the huge state space, we employ the deep Q network (DQN) [26], which combines Q-learning algorithm with deep CNNs, to find an optimal control policy of traffic signals. Deep neural networks (DNNs), including deep CNNs, can automatically learn efficient features from raw and high-dimensional inputs, such as the traffic state defined by event data in this paper, using a general-purpose learning procedure [27,28]. In this study, the enhanced deep Q network with ideas of double Q learning [29] and dueling network architecture [30] is adopted. It is known as double dueling deep Q network (3DQN).

### 4.1. Double Deep Q Network Algorithm

Supposing the optimal action-value function $q^{*}$ is available, the optimal state-value function $v^{*}$ and the optimal action policy $\pi^{*}$ can be easily formed [31] by:

(6) $$\begin{array}{cc} v^{*}\left(s\right) & =\max_{a\in A_{s}}q^{*}\left(s,a\right),\forall s\in S\\ \pi^{*}\left(s\right) & =\underset{a\in A_{s}}{\mathrm{argmax}}q^{*}\left(s,a\right),\forall s\in S \end{array}$$

Therefore, a parameterized CNN, donated as Q(s,a;θ), is employed to directly estimate $q^{*}$, $Q\left(s,a;\theta \right)\approx q^{*}\left(s,a\right)$, where θ is the weights of CNN. Observing state $s_{k}$, the agent takes action $a_{k}$, then, the environment returns reward $r_{k+1}$ and transits to state $s_{k+1}$. This process is recorded as an interaction experience $e_{k}=\left(s_{k},a_{k},r_{k+1},s_{k+1}\right)$. Using $e_{k}$, the network Q(s,a;θ) adjusts its weights θ to approximate an optimal action-value function. A technique named experience replay [32] is employed to handle the problem of learning instabilities. In this method, the interaction experience is stored in a replay memory. When learning, minibatches of experiences *E* are sampled uniformly at random from the replay memory, and θ is updated using the following loss function

(7) $$L\left(\theta \right)=\frac{1}{batch\_size}\sum_{e_{k}\in E}{\left(T_{k}-Q\left(s_{k},a_{k};\theta \right)\right)}^{2}$$

where batch_size is the size of minibatches, $T_{k}$ is the target value, and Adam algorithm [33] is adopted to accelerate training by adjusting learning rate adaptively in this paper. In double DQN, the target value is calculated by

(8) $$T_{k}=r_{k+1}+\gamma Q\left(s_{k+1},\underset{a}{\mathrm{argmax}}Q\left(s_{k+1},a;\theta \right);\theta^{-}\right)$$

where $Q(s,a;\theta^{-})$ is a target CNN network which has the identical structure as Q(s,a;θ), and its weights $\theta^{-}$ is updated using θ periodically. By using the action-value network Q(s,a;θ) and target network $Q(s,a;\theta^{-})$ to select the action and estimate the target value respectively, double DQN can reduce the overestimation bias incurring in DQN, thus result in better performance [29]. After updating θ, we adjust $\theta^{-}$ as follows

(9) $$\theta^{-}=\beta \theta^{-}+\left(1-\beta \right)\theta$$

where β is the target network update rate.

### 4.2. The Deep Convolutional Neural Network

**Figure 4:**
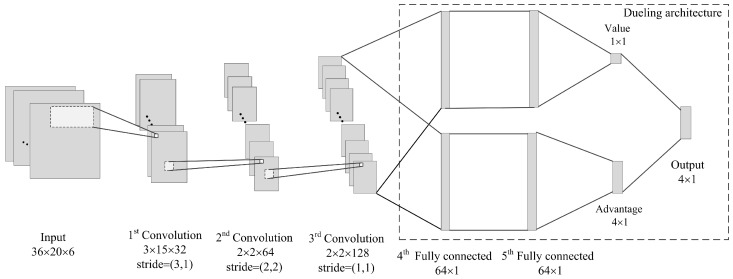
The structure of the deep convolutional neural network.

Considering the characteristics of our state definition, we construct the following CNN network, which is illustrated in Figure 4. The network input has the identical size as the intersection state (it is 36×20×6 at a 4-arms intersection in the paper). The first hidden layer is a convolutional layer, which contains 32 filters of 3×15 with stride of (3,1) and employs a rectifier nonlinearity activation function (ReLU). The second convolutional layer has 64 filters of 2×2 with stride of (2,2) and using a ReLU again. The last convolutional layer contains 128 filters of 2×2 with stride of (1,1) and is also followed by a ReLU. The dueling architecture [30], which estimates the Q value function by combining state-value function and action advantage function, is employed in this network. Therefore, the output data of the last convolutional layer is put through two streams separately, both of which contain two fully connected layers of 64 neurons with ReLUs. The first stream is used to estimate the state value of size 1×1, while the other calculates the advantage of each action. Since 4 actions (phases) are available in this paper, the advantage is of size 4×1. In the last layer, the two streams are combined again to produce the final *Q* value function.

### 4.3. Training Algorithm of Adaptive Traffic Signal

Algorithm 1 presents the training process of the 3DQN-based adaptive traffic signal. Firstly, we initialize variables and parameters, such as the replay memory and weights of action-value network and target network, and obtain the current intersection state and signal phase (line 1–5). Then, the training process, which is composed of selecting action, executing action, observing the environment, storing experience, and adjusting CNN, is iterated until the learning end: 

**Algorithm 1:** Double Dueling Deep Q Network for Traffic Signal Control
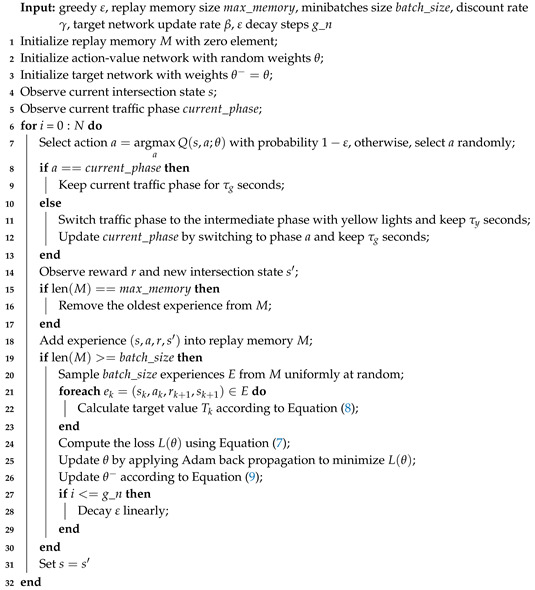

- Action selection: the ε-greedy method is adopted to select the action, i.e., under the probability of ε the action is selected randomly, otherwise, the action with the greatest action-value is chosen (line 7), and ε is decayed linearly from the initial value to the final value over the given steps (line 27–29);
- Action execution: if the action is to keep the current signal phase, prolonging this phase for $\tau_{g}$ seconds. Otherwise, following an intermediate phase of $\tau_{y}$ seconds which indicates yellow signals for the running vehicles, the action (signal phase) is executed for $\tau_{g}$ seconds (line 8–13);
- Observation: After executing the action, the agent observes and obtains the reward and new intersection state (line 14);
- Experience storage: adding the interaction experience produced at this step into the replay memory, which has a limited capacity (line 15–18);
- Network training: when the cumulative experience reaches the requirement of minibatches update, the agent adjusts the weights of action-value network θ via three sub-steps. (1) randomly drawing experiences from the replay memory to form the minibatches of samples (line 20); (2) calculating the target value for each sample in the minibatches based on double networks, where the action-value network is applied for evaluating actions and the target network is used to estimate the target value (line 21–23); (3) performing a back propagation using Adam algorithm with the aim of minimizing the mean squared error (MSE) of minibatches, thus updating θ and $\theta^{-}$ (line 24–26).

## 5. Simulation Experiments

We carry out the experiments based on an open source microscopic traffic simulator SUMO [34]. We obtain the ’real-time’ traffic information (e.g., the event data) and manipulate simulated objects (e.g., traffic signals) in SUMO via Traffic Control Interface (traci). Keras and TensorFlow Python libraries are used to implement the deep reinforcement learning-based traffic signal controller.

### 5.1. Experimental Settings

As is shown in Figure 2a, a 4-arms intersection is under consideration. Each road has three lanes, the left-most lane and the middle lane allows left turning and through movements respectively, and the right-most lane allows both right turning and through movements. Each lane is 300 m in length, we set L0=51 meters and L1=2 meters in Figure 2a. Four signal phases are defined as shown in Figure 2b, $\tau_{g}$ is set to 4 s and $\tau_{y}$ is set to 4 s. We set the reward factor $sf_{t}=sf_{w}=1.8$ for phase 1 and phase 3, and $sf_{t}=sf_{w}=1.0$ for phase 2 and phase 4, and trade-off coefficients $\alpha_{0}=\frac{1}{12},\alpha_{1}=\frac{7}{60}$. Regarding the vehicular demand, the length and minimal gap of vehicles are set to 5 m and 2 m, respectively. The default car-following model in SUMO (i.e., Krauss Model) is employed, where the minimum time headway is set to 1 s, the acceleration and deceleration of vehicles are 0.8 m/s${}^{2}$ and 4.5 m/s${}^{2}$ respectively, and the maximum speed is 15 m/s (i.e., 54 km/h). Vehicular arrivals follow the Poisson process. As is listed in Table 1, average traffic volumes of movements are changed every 15 min to reflect the time-variant traffic demand, totally 6 time periods are considered.

We train the agent for 1000 episodes, each episode corresponds to a simulation of 1.5 h, in which the first 120 s is used to warm up and obtain the initial intersection state. The values of hyper-parameters in the deep reinforcement learning algorithm are shown in Table 2.

In the process of training, the cumulative reward value and queue length in each episode are used to quantify the control policy of the agent. To demonstrate the advantage of event data, we compare our agent to the agent using aggregated traffic data, where the average occupancy and speed collected by detectors d1 and d2 in previous 30 s along with the current signal phase are used to define the traffic state. Considering the relatively few elements in the aggregated traffic state (50 variables), a deep CNN, which consists of two convolutional layers (64 filters of 2×2 with stride of (2,2) and 128 filters of 2×2 with stride of (1,1), followed by a ReLU respectively) and several fully connected layers with the same dueling architecture as in Section 4.2, is employed to extract its features. The aggregated data-based agent (ABA) is trained using the same reinforcement learning method and hyper-parameters as the event data-based agent (EBA).

To evaluate the proposed method, we benchmark the trained agent against two commonly used traffic signal control strategies: optimal fixed-time signal control, whose plan is set using Webster method [21] based on the average flow rates over the whole simulation period, as well as the fully actuated signal control [23]. Table 3 presents the parameters used in them. Five performance metrics, i.e., the vehicle throughput (veh), total delay per vehicle (sec/veh), queue length at the intersection (veh), vehicle speed (km/h), and number of stops are employed to evaluate these methods. All simulations are run 5 times with different random seeds and the average results are presented.

### 5.2. Training Results

**Figure 5:**
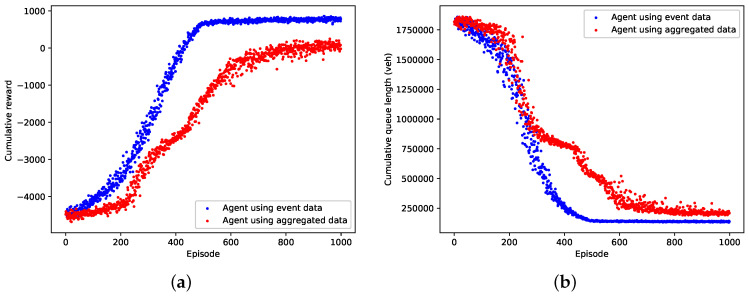
Training performance of the event data-based agent against the aggregated data-based agent. (**a**) Cumulative reward; (**b**) Cumulative queue length.

The learning performance of the event data-based agent and the aggregated data-based agent in terms of cumulative reward and queue length in an episode are shown in Figure 5a,b respectively. From the figures we can see that both the event data-based agent and the aggregated data-based agent can converge to a local optimal control policy by using the deep reinforcement learning technique. Compared with the aggregated data-based agent, the event data-based agent converges faster and learns a better optimized policy which results in greater reward value and fewer queuing vehicles in an episode. Table 4 compares the performance of the event data-based agent and the aggregated data-based agent over the last 100 training episodes. It clearly reveals that the event data-based agent is thoroughly superior to the aggregated data-based agent, as it achieves the improved average performance and lower variance. As is expected, our agent gains conspicuous optimality and stability by exploiting the high-resolution event data.

### 5.3. Evaluation Results

**Figure 6:**
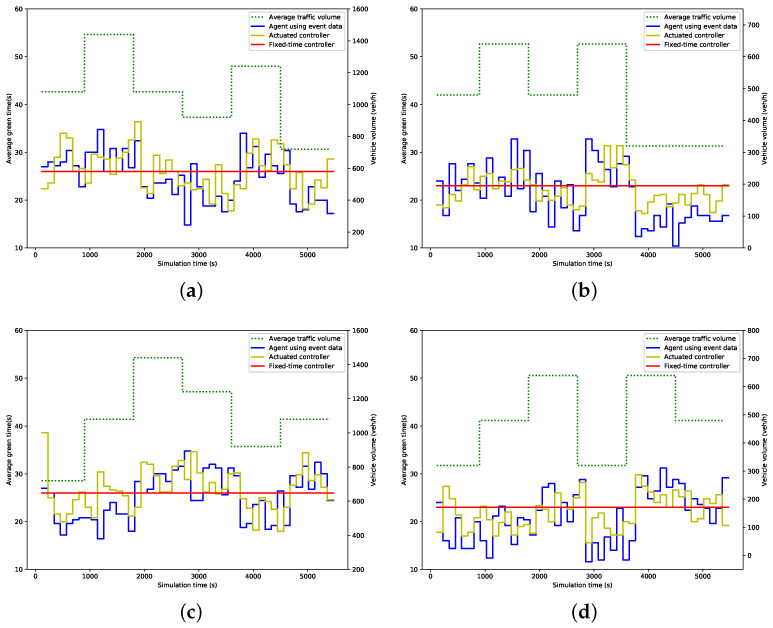
Average green time; (**a**) Phase 1; (**b**) Phase 2; (**c**) Phase 3; (**d**) Phase 4.

**Figure 7:**
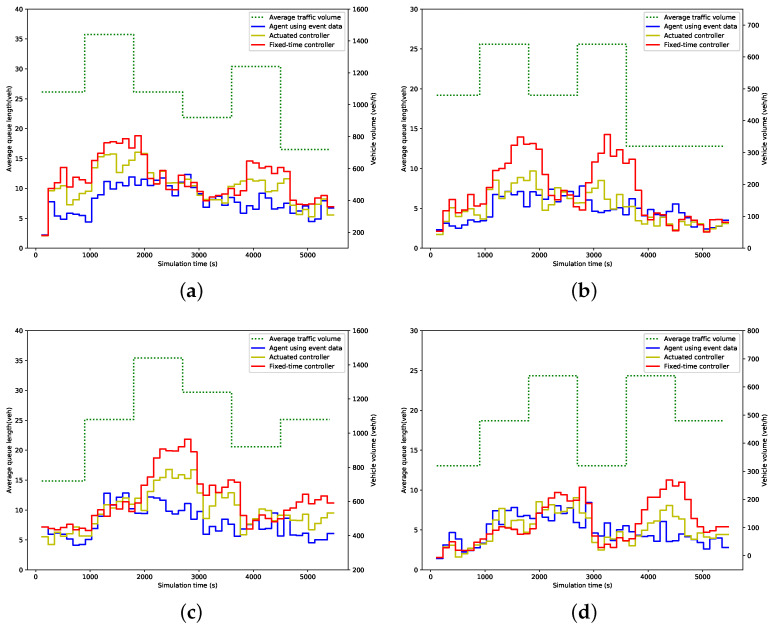
Average queue length; (**a**) Phase 1; (**b**) Phase 2; (**c**) Phase 3; (**d**) Phase 4.

Figure 6 and Figure 7 show the average green time and queue length within 114 s (i.e., a cycle time of the fixed-time controller) for each phase under different control strategies. In these figures, the dot line represents the traffic volume, while the solid lines represent the measured value. As expected, the green time allocated by the proposed agent and actuated controller fluctuates in line with the change of traffic volume, while the fixed-time controller remains at the preset value. Consequently, the fixed-time controller results in more queuing vehicles when traffic volume increases, while the queue length produced by the other two strategies, especially by our agent, is much more steady than that by the fixed-time controller, as is illustrated in Figure 7.

**Figure 8:**
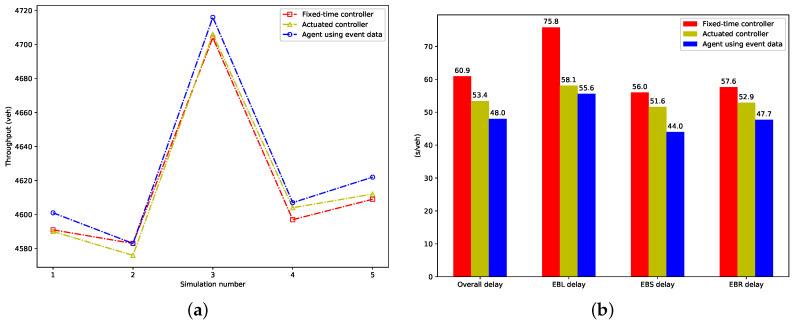
The performance of metrics optimized by the proposed agent; (**a**) Throughput in evaluation simulations; (**b**) Total delay per vehicle.

We calculate the vehicle throughput in each evaluation simulation and total delay per vehicle for each control method, which represent the objectives optimized by the proposed agent, and show them in Figure 8. From Figure 8a, we can see the proposed agent achieves more throughput than the fixed-time and actuated strategy in almost all evaluation simulations. Figure 8b presents the vehicular delay, which includes the overall vehicular delay, delays of EBL (EastBound Left-turning vehicles), EBS (EastBound Straight vehicles), and EBR (EastBound Right-turning vehicles). The proposed agent results in the smallest overall, EBL, EBS, and EBR delay. Compared to the fixed-time controller, a reduction of 21.2%, 26.6%, 21.4% and 17.1% is achieved, respectively. A reduction of 10.1%, 4.3%, 14.7% and 9.8% is achieved respectively, when compared to the actuated controller.

Table 5 lists the quantitative evaluation results. As we can see, the trained agent outperforms the other two control strategies in terms of almost all given metrics in the experiments. It leads to the smallest delay, shortest queue length, and highest vehicle speed with the lowest variances among these three strategies. The advantage of our agent is especially evident for the metric of queue length, 27.9% and 16.4% reduction in queue length is respectively achieved when compared with the fixed-time and the actuated controller. The proposed agent produces slightly more stops than the actuated strategy, while it decreases the number of stops by 5.6% compared with the fixed-time strategy.

## 6. Conclusions

In this paper, we proposed a discrete time traffic state encoding method to define the traffic state using the informative event data, which can be collected directly using prevailing detectors, for designing a reinforcement learning-based traffic signal control system. A double dueling deep Q network is employed to automatically learn useful features from the large-scale and raw state data.

We trained our agent at a simulated 4-arms intersection and compared its training performance against the aggregated traffic data-based agent, the results confirmed that benefiting from the event data, our agent is notably superior to the agent based on the aggregated traffic data in both optimality and stability. Using the trained controller, we benchmarked against two popular signal controllers, the fixed-time controller optimized by Webster method and the actuated controller, under variable traffic demands. The results indicate that our controller achieves the most vehicle throughput and outperforms the fixed-time and the actuated controller by 21.2% and 10.1% in average vehicle delay, 29.7% and 16.4% in queue length, and 15.5% and 6.9% in average vehicle speed, respectively.

Further research will include conducting more comprehensive comparisons by considering other adaptive control methods, performing robustness analyses to disturbances, such as the detector noise, traffic accidents and bad weather conditions, in order to deploy the proposed system at real-world intersections, and developing a coordination algorithm among the proposed RLATC agents to improve the performance of traffic networks further.

## Figures and Tables

**Table 1:** The variable traffic volume (vehicle/hour).

Time (minute)	EW				NS		
	Right Turn	Through	Left Turn		Right Turn	Through	Left Turn
1~15	180	360	240		120	240	160
15~30	240	480	320		180	360	240
30~45	180	360	240		240	480	320
45~60	180	280	320		180	440	160
60~75	180	440	160		180	280	320
75~90	120	240	160		180	360	240

**Table 2:** Hyper-parameters used in the deep reinforcement learning algorithm.

Hyperparameter	Learning rate	Discount factor	Initial ε	Final ε
Value	0.0002	0.75	1.0	0.01
Hyperparameter	ε decay steps	Minibatchs size	Replay memory size	Target network update rate
Value	450,000	32	100,000	0.001

**Table 3:** Parameters of the benchmarked signal control strategies.

		Phase 1	Phase 2	Phase 3	Phase 4
Fixed-time	Green time splits (s)	26	23	26	23
	Cycle length (s)	114			
Fully actuated	Minimum green time (s)	17	17	17	17
	Maximum green time (s)	36	32	36	32
	Unit extension (s)	3.5			
	Passage time (s)	3.4			

**Table 4:** Performance comparison of the last 100 training episodes.

Performance Metrics	EBA			ABA	
	Mean	Std		Mean	Std
Cumulative reward	771.6	37.0		52.1	90.1
Cumulative queue length (veh)	137,485.7	2849.4		209,773.87	14,168.4

**Table 5:** Evaluation performance of our agent.

Performance Metrics	Agent Using Event Data			Fixed-Time			Actuated			Improvement	
	Mean	Std		Mean	Std		Mean	Std		vs. Fixed-Time	vs. Actuated
Vehicular delay (s/veh)	48.0	28.0		60.9	33.5		53.4	29.9		21.2%	10.1%
Queue length (veh)	26.0	7.8		31.0	8.3		36.9	10.0		29.7%	16.4%
Vehicle speed (km/h)	24.5	2.1		22.9	2.3		21.2	4.2		15.5%	6.9%
Number of stops (#/veh)	0.85	0.42		0.90	0.41		0.83	0.40		5.6%	−2.2%
